# Supplementary material for: Osteoarthritis as a Systemic Disease Promoted Prostate Cancer In Vivo and In Vitro
Source: Int J Mol Sci. 2024 May 30;25(11):6014. doi: 10.3390/ijms25116014 (PMC11172560; doi:10.3390/ijms25116014)
Supplement: Supplementary file 1 [file ijms-25-06014-s001.zip › ijms-3019401-supplementary.pdf]

International Journal of
Molecular Sciences

MDPI

Supplemental Data

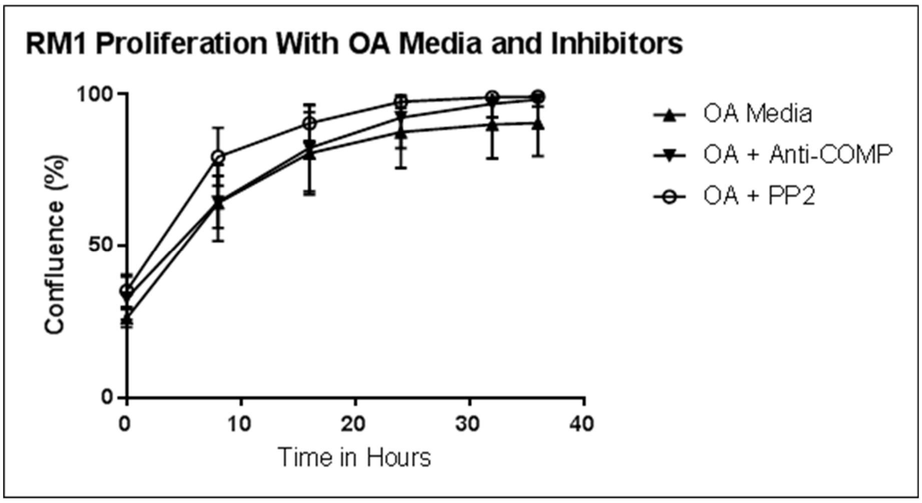

Figure S1. Evaluating whether inhibition of COMP or some downstream pathways would significantly alter the growth rate of the cells, we saw no significant differences in the growth rates of RM1 cells treated with OA media vs. OA media plus inhibitors (p=0.836, linear regression).

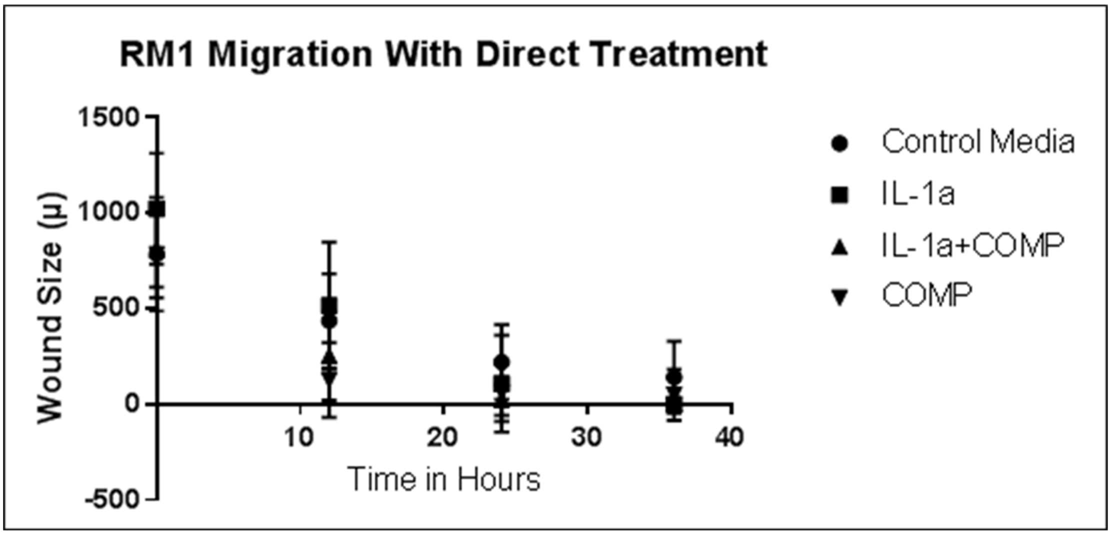

Figure S2. Evaluating the effects of direct application of IL-1α and/or with COMP we found no increased rate of migration with the scratch wound assay (p=0.650) amongst the studied groups.

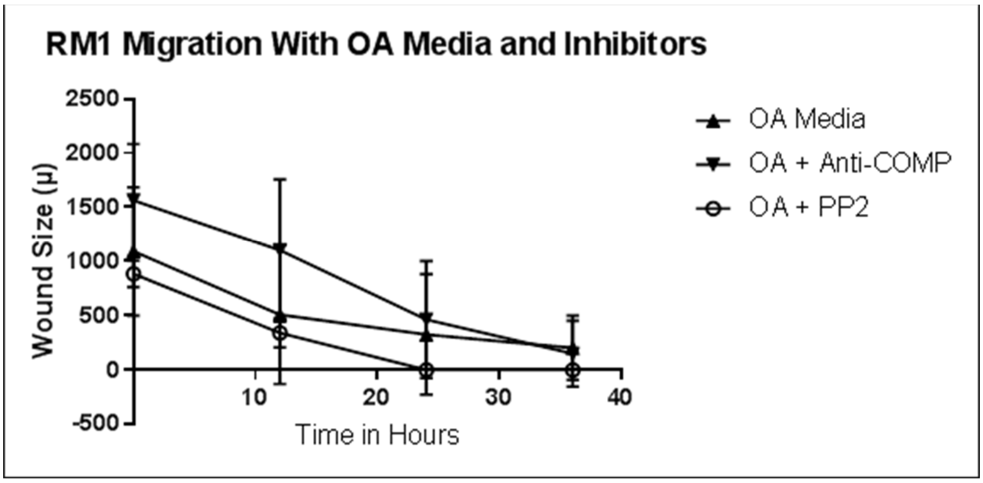

Figure S3. Later, when evaluating whether adding inhibitors to the conditioned media altered this relationship, we found that various COMP inhibitors were unable to lead to a significant difference vs. OA media alone (p=0.246, linear regression).

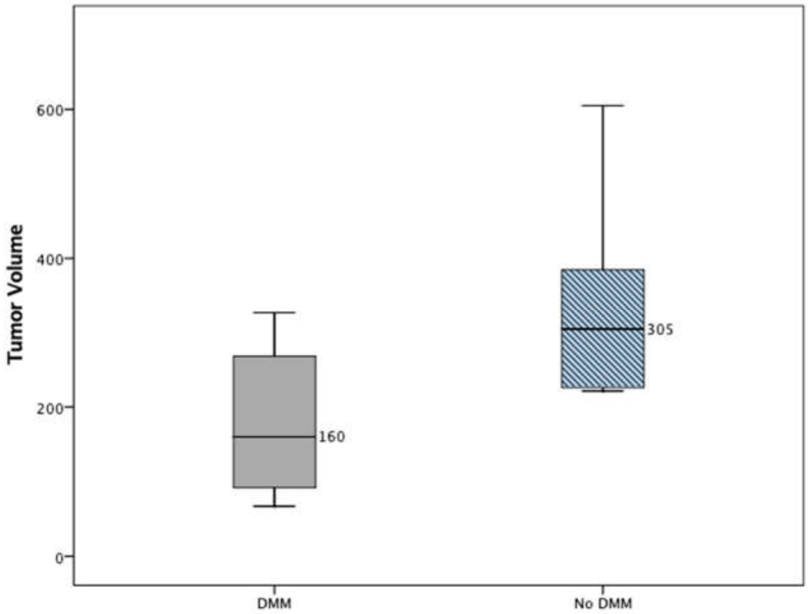

Figure S4. Tumor volume calculations at time of euthanasia as allocated by group with no significant difference noted (Mann Withney U test, p=0.650).

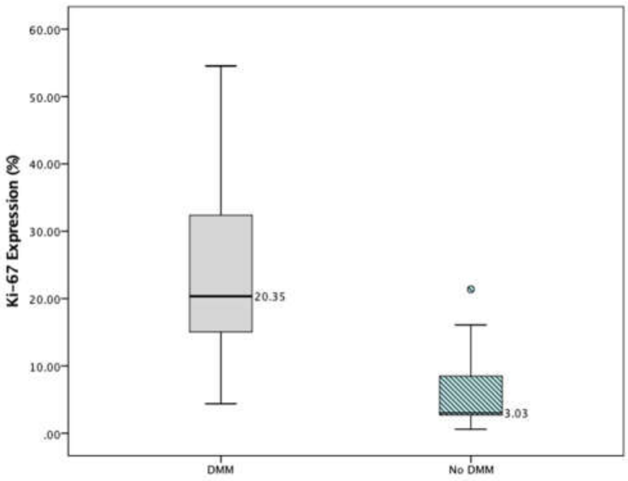

Figure S5. Distribution and comparison of Ki-67 expression percentage according to group allocation (p=0.004, unpaired t-test)

Table S1. Results of IHC. Unpaired t-tests performed.

**IHC results**	**PSMA %**		**COMP %**		**CD-31 %**	
	**DMM**	**No DMM**	**DMM**	**No DMM**	**DMM**	**No DMM**
	1.86	0.15	11.44	0.78	5.59	14.49
	2.72	1.58	0.56	1.26	62.62	10.59
	0.14	0.59	4.29	0.32	6.33	15.39
	1.25	2.12	6.33	13.51	1.51	18.92
	1.95	1.07	4.09	3.59	11.55	85.25
	2.13	7.18	22.42	28.16	49.39	70.78
	2.83	5.73	4.09	4.30	43.70	14.24
	0.14	1.99	0.37	0.16	4.26	0.43
	5.09		11.18		2.55	14.93
	0.44		5.24		5.42	
	3.19		8.10		16.54	
**Mean**	1.98	2.55	7.10	6.51	19.04	27.22
**Median**	1.95	1.78	5.24	2.43	6.33	14.93
**SD**	1.49	2.53	6.24	9.78	21.95	29.47
**Statistical Comparison**	p=0.541		p=0.342		p=0.485	
